# Differences in Hemodynamic Response to Passive Leg Raising Tests during the Day in Healthy Individuals: The Question of Normovolemia

**DOI:** 10.3390/life13071606

**Published:** 2023-07-21

**Authors:** Andrius Pranskunas, Egle Gulbinaite, Aiste Navickaite, Zivile Pranskuniene

**Affiliations:** 1 Department of Intensive Care Medicine, Lithuanian University of Health Sciences, Eiveniu g. 2, 50161 Kaunas, Lithuania; 2 Faculty of Medicine, Medical Academy, Lithuanian University of Health Sciences, A. Mickeviciaus g. 9, 44307 Kaunas, Lithuania; egle.gulbinaite@stud.lsmu.lt (E.G.); aiste.navickaite@stud.lsmu.lt (A.N.); 3 Institute of Pharmaceutical Technologies, Lithuanian University of Health Sciences, Sukileliu pr. 13, 50166 Kaunas, Lithuania; zivile.pranskuniene@lsmu.lt; 4 Department of Drug Technology and Social Pharmacy, Lithuanian University of Health Sciences, Sukileliu pr. 13, 50166 Kaunas, Lithuania

**Keywords:** passive leg raising, circadian rhythms, preload responsiveness, normovolemia

## Abstract

Background: The passive leg-raising (PLR) test was developed to predict fluid responsiveness and reduce fluid overload. However, the hemodynamic response of healthy individuals to the PLR test and how it changes during the day, between the morning and evening, after individuals have consumed food and fluids, has not been profoundly explored. This study aimed to compare the systemic hemodynamic changes in healthy individuals between morning and evening PLR tests. Methods: In this study, the PLR test was performed twice a day. The first PLR test was performed between 08h00 and 09h00 in the morning, while the second PLR test was performed between 20h00 and 21h00 in the evening. Hemodynamic parameters were measured using an impedance cardiography monitor, and a cutoff value of a 10% increase in stroke volume (SV) during the PLR test was used to differentiate between preload responders and non-responders. Results: We included 50 healthy volunteers in this study. When comparing the morning and evening PLR test results, we found no PLR-induced differences in heart rate (−3 [−8–2] vs. −2 [−8–4] beats/min, *p* = 0.870), SV (11 [5–22] vs. 12 [4–20] mL, *p* = 0.853) or cardiac output (0.7 [0.2–1.3] vs. 0.8 [0.1–1.4] L/min, *p* = 0.639). We also observed no differences in the proportion of preload responders during the PLR test between the morning and evening (64% vs. 66%, *p* = 0.99). However, there was a moderate agreement between the two PLR tests (morning and evening) (kappa = 0.429, *p* = 0.012). There was a moderate correlation between the changes in SV between the two PLR tests (r_s_ = 0.50, *p* < 0.001). Conclusion: In young, healthy individuals, we observed no change in the systemic hemodynamic responsiveness to the PLR test between the morning and evening, without restriction of fluid and food intake.

## 1. Introduction

Recently, there has been increasing interest in passive leg raising (PLR) as a simple method to induce physiological perturbation and measure cardiovascular responses. It is a bedside test used to assess fluid responsiveness [1,2] and baroreceptor function [3], detect subclinical left ventricular dysfunction [4], and measure arterial vasodilator reserve and endothelial function [5]. This test involves raising the legs of the patient to a 45° angle, which induces a sudden increase in preload due to the autotransfusion of blood from the venous reservoir of the lower extremities to the central venous compartment, leading to an increase in stroke volume (SV) and cardiac output (CO) in preload-dependent patients. When PLR is initiated from the supine position, its increasing effect on cardiac preload is lower than when it is initiated from the semi-recumbent position [6]. Therefore, the standard starting position of PLR is the semi-recumbent position. In critically ill patients with hypotension, PLR is often used to predict fluid responsiveness and determine the need for fluid infusion [7]. In fact, approximately 50% of patients with hemodynamic instability are fluid responders [8]. This means that, according to Frank-Starling’s law, the heart can increase SV or CO by more than 10–15% in response to volume loading [9,10]. This concept of fluid responsiveness implies that fluids are administered to patients with shock only when they are fluid responders, thereby preventing harm as a result of fluid administration (e.g., pulmonary edema). A previous meta-analysis has shown that fluid overload and a positive cumulative fluid balance are associated with increased mortality in critically ill patients [11]. Therefore, it is important to ask whether critically ill patients need to be completely volume-filled to make them non-responders. The response to PLR in healthy subjects may help answer this question.

Although PLR has been found to increase preload and stroke volume and help predict fluid responsiveness in critically ill patients with hypotension [12], the hemodynamic effects of PLR in normotensive patients and healthy subjects are unclear. Using the PLR test, a previous study showed that approximately 50% of healthy volunteers were fluid responders [13]. However, the response to PLR changes after consuming the natural daily amount of fluids or food remains unknown. Moreover, it is unclear whether healthy individuals become non-responders after daily fluid intake. In contrast, it is known that the circadian system affects cardiovascular responses to postural stress, resulting in a greater susceptibility to presyncope during the night or morning [14].

Therefore, we aimed to compare the systemic hemodynamic changes in healthy individuals between PLR testing in the morning and evening, after routinely consuming food and liquids.

## 2. Materials and Methods

### 2.1. Subjects

We included healthy, recreationally active volunteers, aged 18 years or above in this prospective cohort study. The exclusion criteria included pregnancy, any acute illness, identified cardiovascular disease, or known risk of thromboembolism. The participants were instructed not to consume caffeine, alcohol, tobacco, or engage in physical activity in the morning prior to the measurements. 

### 2.2. Protocol

**Figure 1:**
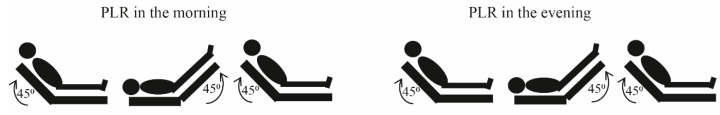
Overview of the study procedure. PLR, passive leg raising.

The PLR test was performed twice a day. The first PLR test was performed between 08h00 and 09h00 in the morning, and the second was performed between 20h00 and 21h00 in the evening (Figure 1). In the evening, the approximate amount of fluid consumed, the number of cups of coffee, and physical activity were recorded.

We measured hemodynamic parameters using an impedance cardiography (ICG) monitor (Niccomo; Medis, Ilmenau, Germany). ICG has been widely compared to different invasive and noninvasive methods and has been validated for healthy individuals at rest and during exercise [15,16], patients undergoing surgery [17], and individuals with cardiovascular diseases [18].

For SV and CO measurements, four electrodes were placed on the neck and thorax as follows, according to the manufacturer’s instructions: two electrodes on the left side of the neck and two electrodes on the left side of the lower chest. The SV was calculated beat by beat and averaged over 16 heartbeats.

The subjects were placed in a semi-recumbent position for at least 5 min, with the backrest of the bed folded to form a 45° angle. Subsequently, we measured the baseline hemodynamic parameters. This was followed by a PLR maneuver (Figure 1), in which the body was moved to a supine position and both legs were raised 45° from the bed for 2 min. The highest SV value was recorded during the PLR. We used a cutoff value of a 10% increase in SV during PLR to differentiate between preload responders and non-responders. After completing the PLR test, the participant was returned to a semi-recumbent position for 5 min. This was followed by the measurement of hemodynamic parameters. 

### 2.3. Statistical Analysis

In this study, the primary outcome was the percentage of subjects in whom a PLR test resulted in a ≥10% improvement in noninvasively measured SV, as well as the correlation between the changes in SV during PLR in the morning and evening.

We calculated the sample size based on the SV from our observations and prior trials with similar populations [13,19]. Assuming a mean SV of 80 mL in the semi-recumbent position, we estimated that at least 39 patients were required to detect a clinically relevant difference of 8 mL (10% increase) in SV after PLR, with a power of 90% and an alpha of 0.05. Furthermore, our study was powered to detect at least a moderate correlation (Spearman correlation coefficient ≥0.5, minimum of 43 participants required) between the changes in SV during PLR in the morning and evening, with 90% power associated with a two-sided alpha of 0.05. 

Statistical analysis was performed using SPSS software (IBM Corp., version 27, New York, NY, USA). We used the Shapiro–Wilk test to check the data for normality, and data with a non-normal distribution are presented as medians with interquartile ranges. Using the Wilcoxon test, we tested differences between the morning and evening PLR tests. Additionally, we used the Friedman test to compare multiple sets of related values, followed by the Wilcoxon test with the Bonferroni correction for multiple pair comparisons. Furthermore, McNemar’s test was used to determine any differences in the dichotomous dependent variables, such as preload responsiveness, between the two related groups. Agreement in determining preload responsiveness between two PLR tests (morning and evening) was tested using Cohen’s Kappa. Correlations were tested using Spearman’s correlation test, and statistical significance was set at *p* < 0.05.

## 3. Results

We included 50 healthy individuals (34 females and 16 males; median age 23 years) (Table 1).

**Figure 2:**
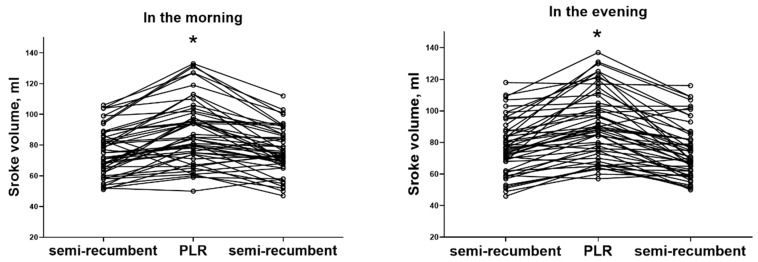
Individual stroke volume changes during passive leg raising (PLR) in the morning and evening. * *p* < 0.05.

In the semi-recumbent position, the subjects had a median SV of 74 (64–87) and 77 (62–87) mL before PLR in the morning and evening, respectively, which were not significantly different (*p* = 0.186). In contrast, PLR significantly reduced heart rate (HR) and increased SV and cardiac output (CO) both in the morning and evening (Table 2, Figure 2). However, we found no PLR-induced differences in HR (−3 [−8–2] vs. −2 [−8–4] beats/min, *p* = 0.870), SV (11 [5–22] vs. 12 [4–20] mL, *p* = 0.853), or CO (0.7 [0.2–1.3] vs. 0.8 [0.1–1.4] L/min, *p* = 0.639) when comparing the morning and evening values. Accordingly, we found no differences in changes expressed as percentages in SV (14 [6–33] vs. 19 [5–28]%, *p* = 0.858), or CO (14 [3–24] vs. 12 [2–26]%, *p* = 0.783) when comparing the morning and evening.

Despite the variations in HR and CO, the transition to a semi-recumbent position after PLR resulted in the SV returning to baseline in both the morning and evening tests (Table 2, Figure 2). Moreover, we found a significant negative correlation between the changes in HR and SV both in the morning PLR test (r_s_ = −0.46, *p* < 0.001) and the evening PLR test (r_s_ = −0.36, *p* = 0.01).

In contrast, we found no difference in the changes in mean arterial pressure or systemic vascular resistance index in the semi-recumbent position before and after PLR when we compared morning and evening values. However, we observed a significant increase in baseline mean arterial pressure and CO in the evening when compared to the morning (Table 3).

### Preload Responsiveness

**Figure 3:**
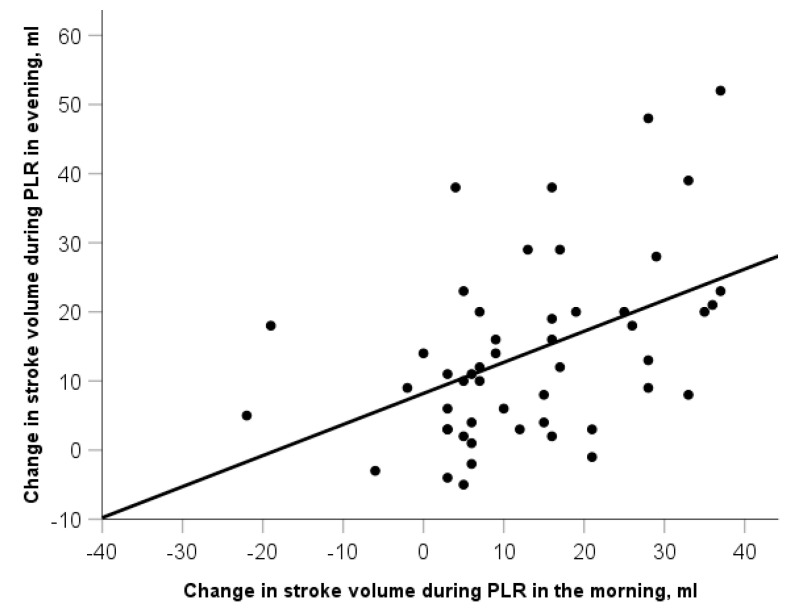
Correlation between the changes in stroke volume during passive leg raising (PLR) in the morning and evening: r = 0.5, *p* < 0.001.

We found no differences in the proportion of preload responders (SV change ≥10%) during PLR between the morning and evening tests (64% vs. 66%, *p* = 0.99). However, the agreement between the two measurements (morning and evening) was moderate (kappa = 0.429, *p* = 0.012). The changes in SV in the two PLR tests (morning and evening) were moderately correlated (r_s_ = 0.50, *p* < 0.001) (Figure 3).

Preload responders, who had a ≥10% increase in SV during PLR, had a median ∆SV of 26 (15–39)% in the morning and 25 (19–35)% in the evening. In contrast, the median ∆SV for non-responders was 5 (−1–7)% in the morning and 3 (−2–5)% in the evening. Notably, the ∆HR was similar for both responders and non-responders in the morning and evening (responders: −6 [−12–1]% vs. −5 [−14–2]%, non-responders: 2 [−6–7]% vs. 0 [−6–13]%).

Furthermore, when comparing baseline characteristics, baseline SV, CO, and HR did not significantly differ between responders and non-responders, both in the morning and evening (Table 1). We also found no differences in age or body mass index between responders and non-responders in either the morning or evening.

Participants consumed a median of 1000 (550–1400) mL of fluid orally. Forty-two participants (84%) also drank a median of two cups of coffee. We found no correlation between changes in SV during the evening PLR and the amount of fluid or coffee cups consumed during the day. We also found no differences in the amount of fluid consumed during the day between preload responders and non-responders (800 (500–1500) vs. 1000 (750–1000) mL, *p* = 0.564). During the day, 47 (94%) subjects walked or engaged in nonintensive physical activities. Only three subjects attended a sports club.

## 4. Discussion

To our knowledge, this is the first study to examine the differences in hemodynamic responses to PLR in the morning and evening. Until now, the normal response to PLR in healthy individuals was not clear. 

In critically ill patients, the hemodynamic response to PLR helps identify responders and thus decides whether additional fluid infusion can be performed [20,21]. However, there is currently a dilemma regarding whether all critically ill preload responders require fluid infusion until they become non-responders. First, we decided to investigate the normovolemic state in healthy individuals. We investigated whether healthy individuals became non-responders after consuming fluid and food during the day. In this study, we attempted to determine how many preload responders there were among healthy individuals and how the hemodynamic responses changed from the morning to the evening after the subjects consumed food and fluids during the day. Notably, we found no significant difference in hemodynamic changes during PLR between the morning and evening tests. Additionally, the proportion of responders in the morning and evening remained similar, at approximately 60%. 

Regarding this proportion of responders, our results were similar to those of a previous study that used the same hemodynamic monitor [13]. In that study, the researchers performed two consecutive PLR tests and identified approximately 50% of the responders using an SV change of ≥10% to define a positive response, which showed good categorical agreement. In their study, the ΔSV in the two PLR tests was strongly correlated (rs = 0.68). However, they did not state the times of day that the PLR tests were performed. In our study, we found a moderate correlation between the ΔSV in the morning and evening, which may indicate physiological differences related to the time of day. Interestingly, another study compared hemodynamic changes on the PLR test in the morning when subjects were fasting or had eaten a normal breakfast. In this study, 10 healthy participants came to the laboratory in the morning at two time points at least 24 h apart. In addition, participants were randomized to either fasting or a normal breakfast with fluids and food (control). At both visits, participants underwent PLR. In this study, no differences in ΔSV were observed [22]. The total water deficit after 6–8 h of fasting was about 500 mL [23,24]. In another study, it was found that preoperative fasting did not change dynamic and static preload indices [25]. Our study participants consumed a median of 1000 mL of fluid and 2 cups of coffee per day.

Other previous studies on healthy individuals using different hemodynamic monitoring methods have shown a responsiveness of approximately 44–96% to PLR at a 10–15% SV or CO threshold [13,19,26,27]. However, compared to these studies, our study had one of the largest sample sizes. Interestingly, a previous systematic review showed that the rate of fluid responsiveness in emergency department patients ranged from 31–79% [28]. Additionally, a meta-analysis of studies evaluating PLR in intensive care unit patients demonstrated a 54 ± 9% positive response rate to fluid challenges [9]. In critically ill patients who responded to fluid infusion, PLR resulted in a mean CO change of 20 ± 9% [9] and the same change in SV [29,30]. In our study with healthy subjects, the change in SV of PLR responders had similar values. Another study with healthy volunteers also showed similar results [13].

During PLR, approximately 250–350 mL of venous blood was transferred from the lower body toward the right heart, without fluid infusion [2,10]. Thus, PLR mimics a fluid challenge and helps prevent dangerous side effects of fluid overload by avoiding fluid infusion in non-preload responders. Methodical PLR performance is important for accurate results. The starting position for PLR must be semi-recumbent. The study has shown that PLR in a semi-recumbent position, in contrast to a supine position, leads to additional redistribution of venous blood to the lower part of the body and increases the PLR effect by increasing SV and CO [6]. The reliability of PLR is best when the effects are measured by a direct measure of SV or CO [9,10]. We used the ICG method, which provides a true continuous beat-to-beat measurement of SV. ICG is a noninvasive, operator-independent method of measuring impedance changes caused by a specific current flowing through the chest, using four electrodes. These electrodes also record the ECG signal to detect the cardiac cycle. Thus, this technology measures changes in aortic flow and blood volume that affect changes in thoracic impedance [31]. ICG has been validated in healthy volunteers [15,16] and under certain clinical conditions [17,18]. However, inaccurate and unreliable results may occur with the ICG technique due to motion artifacts, arrhythmias and tachycardias, excessive thoracic fluid, and severe valvular disease [31].

In a clinical setting, a PLR-induced increase in SV or CO of more than 10–15% reliably predicts fluid response in patients with spontaneous breathing [32]. Monnet and Teboul recommend measuring cardiac output continuously during the first minute of elevation, since the effects of PLR may disappear after one minute. In this regard, there are mixed data in the literature, usually covering an observation window of 1 to 3 min [6,9,19,27]. In our study, we elevated the legs for a 2 min observation window to ensure that all subjects reached the maximum SV. In addition, studies with young, healthy subjects have shown that the maximum increase in SV is achieved in a 1–2 min period with legs elevated [13]. After 7–10 min of elevating the legs, this effect of increased SV disappears [33,34]. Thus, the effect is not sustained, and SV returns to the initial level after returning to the initial position. If the PLR test is performed correctly, SV should return to the baseline after returning to the semi-recumbent position [2]. In our study, we demonstrated that SV returned to the baseline when subjects returned to a semi-recumbent position. Another important methodological aspect of PLR is to avoid additional sympathetic stimulation, which would be indicated by an increase in HR. We found that elevating the legs significantly reduced HR in our study. In this way, we may be able to rule out an external stimulus. However, we recorded HR at the time when the maximum SV was reached. Therefore, we cannot completely rule out other changes in HR at other time points. Studies in healthy subjects have shown that PLR is associated with a counterregulatory autonomic response [35]. Thus, an initial predominance of sympathetic activity in the semi-recumbent position was displaced by a relative increase in parasympathetic activity during PLR in parallel with the increase in SV in response to the increase in preload. 

On the other hand, acute and chronic changes in blood volume in healthy individuals need to be discussed. Changes in PLR with acute blood volume loss are demonstrated in a study by Wong and colleagues [36]. In blood donors, the increase in CI after PLR did not reach the baseline value before blood donation but was also not significantly different. After blood donation, former non-responders became responders (CI increased by 11% during PLR). It should be noted that the initial position of the PLR was supine. However, we did not study the effect of fluid infusion on PLR and how long this effect lasted. Chronic circulatory filling is found in pregnant women in the third trimester. In them, an increase in blood volume by about 50 percent leads to a lack of response [37]. Of course, the presence of increased intra-abdominal pressure must also be considered. Studies in healthy subjects comparing a 6 h fasting with a normal breakfast showed no difference in SV during PLR [22]. 

Hemodynamic response rates are similar in healthy and critically ill patients, and the diurnal rhythm does not alter the response. Therefore, the timing and amount of fluid to be administered to critically ill patients remains unspecified. Furthermore, a complex process related to fluid redistribution occurs during PLR. Hemodynamic changes induced by PLR—when the semi-recumbent position is transferred to a supine position with the legs raised—may simultaneously contribute to cardiovascular changes not only by an increase in cardiac preload as a result of blood autotransfusion from the legs, thus increasing the SV or CO (Frank-Starling law) but also by an autonomic nervous system effect resulting from nociceptive stimulation, activation of the baroreflex [38], and endothelial response to shear stress [5]. These processes may be more pronounced in healthy individuals with an intact autonomic system, endothelium, and organ function. However, in critically ill patients, PLR is closely related to the systemic hemodynamic response to fluid challenges. In fact, a previous meta-analysis showed a pooled sensitivity of 0.85 (0.81–0.88) and a pooled specificity of 0.91 (0.88–0.94) for PLR-induced changes in CO or its surrogate [9]. 

A meta-analysis showed that PLR reliably predicted fluid responsiveness in patients with spontaneous breathing (AUC: 0.74–0.94) [32,39]. PLR has the following limitations: it requires direct measurement of SV or CO, and it cannot be used in patients with intracranial hypertension and intra-abdominal hypertension [39]. Alternatives to PLR may include respiratory variations of inferior vena cava (IVC) (area under the curve [AUC] 0.85) [40]. Deep and standardized breathing and measurement of IVC diameter 4 cm caudal to the IVC-right atrial junction improved the accuracy of the IVC collapsibility index to discriminate fluid responders (AUC 0.89–0.98) [40,41]. Forced [42] deep inspiratory breathing [43] or paced breathing with additional expiratory resistance [44] also demonstrated the improved predictive utility of PPV or SVV for detecting a fluid response during spontaneous breathing (AUC 0.85–0.91). However, these alternatives have not been fully investigated in healthy subjects.

In a previous study, in the context of circadian reactivity to passive postural changes performed on a tilt table, while moving from a supine position to a 60° head-up position for 15 min, presyncope occurred in half of the healthy participants during the circadian phases corresponding to the biological night (22 h 30 pm to 10 h 30 am) [14]. However, in our study, we found no significant hemodynamic changes between the PLR tests in the morning or evening. This may be related to the effect of the volunteers’ daily routine before they arrived at the laboratory. 

In contrast, we observed a significant increase in baseline mean arterial pressure and CO in the evening compared to the morning. However, previous studies have shown no effect of the circadian rhythm on blood pressure, owing to a lack of strict environmental factor control, which confounded the interpretation of the results [45]. Notably, a study on healthy young adults using strict circadian protocol demonstrated that the highest blood pressure occurred at the circadian time corresponding to 21h00 pm, suggesting that the role of circadian rhythm in blood pressure was unlikely to affect the well-documented morning peak in adverse cardiovascular events [45,46]. Another study confirmed these findings in adults with a mean age of 51 years [47]. It is known that blood pressure is the result of the relationship between CO and peripheral vascular resistance, where blood volume, sympathetic activity, the renin-angiotensin-aldosterone system, the hypothalamic-pituitary-adrenal axis, renal function, and endothelial function play important roles [48].

The present study had some limitations. First, the study population comprised young healthy individuals; therefore, the results cannot be extended to general or older populations. Additionally, the sample size in our study was relatively small. However, the sample of our study was one of the largest in similar studies [13,19,26,27]. We did not measure the autonomic function markers (HR variability, epinephrine, norepinephrine, and indices of cardiac vagal tone) that may underlie the circadian rhythm. The change in HR or HR variability are used to reflect a sympathetic tone. The 5% variation in HR can be accepted as a physiological variation in a clinical setting. The large variation of HR, caused by PLR, could indicate a methodologically incorrect PLR, suggesting suprathreshold sympathetic stimulation [49]. We did not measure intra-abdominal pressure. Our subjects were healthy and not overweight, so the possibility of increased intra-abdominal pressure was minimal. PLR was not informative in the presence of elevated intra-abdominal pressure. The study showed that the normal hemodynamic response to PLR in pregnancy was maintained at 22–24 weeks’ gestation [50]. PLR significantly increased SV and decreased blood pressure in both pregnant and nonpregnant women. HR was significantly decreased in pregnant women and did not significantly change in nonpregnant women. However, a study of a cohort of pregnant women in the third trimester showed no significant changes in cardiac output during supine PLR, right lateral decubitus, and left lateral decubitus [37]. This can be explained by compression of the uterus on the inferior vena cava. Thus, obstruction at the level of the inferior vena cava prevents an increase in preload during PLR. Intra-abdominal pressure may vary between 5 and 29 mmHg during pregnancy. It may reach a level of 20 mmHg, which is called abdominal compartment syndrome in severe patients. Another explanation may be related to the complete filling of the circulation when the heart is in the straight part of the curve according to Starling’s law. Then, the pregnant woman is a preload non-responder. It is also known that pregnant women have attenuated baroreflex activity, which increases tolerance to orthostatic stress [51].

## 5. Conclusions

In young, healthy individuals, we observed no change in the systemic hemodynamic responsiveness to the PLR test between the morning and evening, without restriction of fluid and food intake. Furthermore, the proportion of identified preload responders among the healthy subjects was approximately 60%. A similar response rate in critically ill patients has been noted in previously published studies. To answer the question of whether all critically ill patients need to receive fluids until they no longer respond, further studies specifically designed for this patient group are needed.

## Figures and Tables

**Table 1:** Baseline characteristics of the participants.

	All (n = 50)	In the Morning		In the Evening	
		Responders (n = 32)	Non-Responders (n = 18)	Responders (n = 33)	Non-Responders (n = 17)
Gender, female, n (%)	34 (68)	21 (66)	13 (72)	22 (67)	12 (71)
Age, years	23 (22–24)	23 (22–24)	23 (22–23)	23 (23–24)	23 (22–24)
Body mass index, kg/m^2^	21.8 (20.0–23.8)	22.0 (20.2–24.1)	21.5 (19.8–22.7)	21.9 (20.0–24.3)	21.7 (20.1–23.3)
Stroke volume, mL	74 (64–87)	71 (63–87)	76 (65–87)	75 (60–84)	79 (69–98)
Cardiac output, L/min	5.9 (5.3–6.7)	5.9 (4.9–6.5)	6.1 (5.5–6.8)	6.2 (5.3–6.8)	6.8 (5.8–7.5)
Heart rate, beats/min	81 (70–90)	80 (69–90)	83 (70–90)	84 (73–92)	81 (69–96)

**Table 2:** Hemodynamic changes during passive leg raising in the morning and evening.

	In the Morning			In the Evening		
	Before PLR	PLR	After PLR	Before PLR	PLR	After PLR
Heart rate, beats/min	81 (70–90)	78 (69–85) ^a^	74 (64–85) ^ac^	82 (72–92)	81 (70–89) ^b^	79 (68–88) ^b^
Cardiac output, L/min	5.9 (5.3–6.7)	6.6 (5.8–7.6) ^a^	5.4 (4.8–6.3) ^c^	6.3 (5.4–7.1) ^a^	7.2 (6.0–8.1) ^b^	5.6 (5.0–6.4) ^bd^
Stroke volume, mL	74 (64–87)	85 (74–101) ^a^	74 (68–88) ^c^	77 (62–87)	89 (75–106) ^b^	71 (62–86) ^d^

^a^ *p* < 0.001 compared with baseline in the morning; ^b^ *p* < 0.02, compared with baseline in the evening; ^c^ *p* < 0.02 compared with PLR in the morning; ^d^ *p* < 0.02 compared with PLR in the evening; PLR, passive leg raising.

**Table 3:** Hemodynamic parameters of the participants in semi-recumbent positions at rest before and after PLR in the morning and evening.

	In the Morning			In the Evening		
	Before PLR	After PLR	*p*	Before PLR	After PLR	*p*
MAP, mmHg	92 (88–100)	91 (86–97)	0.010	96 (92–103) ^a^	95 (89–101)	0.004
SVRI	2158 (1950–2447)	2221 (1827–2576)	0.696	2157 (1851–2374)	2147 (1964–2562)	0.120

^a^ *p* < 0.01 compared to baseline in the morning; PLR, passive leg raising; MAP, mean arterial pressure; SVRI, systemic vascular resistance index.

## Data Availability

The datasets generated for this study are available upon request made to the corresponding author.
